# A Sustainable Community-Based Model of Noncommunicable Disease Risk Factor Surveillance (Shraddha-Jagrithi Project): Protocol for a Cohort Study

**DOI:** 10.2196/27299

**Published:** 2021-10-22

**Authors:** Jaideep Menon, Mathews Numpeli, Sajeev P Kunjan, Beena V Karimbuvayilil, Aswathy Sreedevi, Jeemon Panniyamakkal, Rakesh P Suseela, Rajesh Thachathodiyil, Amitava Banerjee

**Affiliations:** 1 Amrita Institute of Medical Sciences & Research Centre Amrita Vishwa Vidyapeetham Kochi India; 2 National Health Mission Kochi India; 3 Amrita Institute of Medical Sciences Kochi India; 4 Sree Chitra Thirunal Institute of Medical Sciences and Technology Thiruvananthapuram India; 5 Institute of Health Informatics University College London London United Kingdom

**Keywords:** non-communicable diseases, surveillance, accredited social health activist, panchayat (village), primary health centre, spoke and hub, non-communicable diseases, cardiovascular, public health, hypertension, health services, health center, diabetes

## Abstract

**Background:**

India has a massive noncommunicable disease (NCD) burden, at an enormous cost to the individual, family, society, and health system at large, despite which prevention and surveillance are relatively neglected. If diagnosed early and treated adequately, risk factors for atherosclerotic cardiovascular disease would help decrease the mortality and morbidity burden. Surveillance for NCDs, creating awareness, positive lifestyle changes, and treatment are the proven measures known to prevent the progression of the disease. India is in a stage of rapid epidemiological transition, with the state of Kerala being at the forefront, pointing us towards likely disease burden and outcomes for the rest of the country in the future. A previous study done by the same investigators in a population of >100,000 revealed poor awareness, treatment of NCDs, and poor adherence to medicines in individuals with CVD.

**Objective:**

This study aimed at assessing a sustainable, community-based surveillance model for NCDs with corporate support fully embedded in the public health system.

**Methods:**

Frontline health workers will check all individuals in the target group (≥age 30 years) with further follow-up and treatment planned in a “spoke and hub” model using the public health system of primary health centers as spokes to the hubs of taluk or district hospitals. All data entry done by frontline health workers will be on a tablet PC, ensuring rapid acquisition and transfer of participant health details to primary health centers for further follow-up and treatment.

**Results:**

The model will be evaluated based on the utilization rate of various services offered at all tier levels. The proportions of the target population screened, eligible individuals who reached the spoke or hub centers for risk stratification and care, and community-level control for hypertension and diabetes in annual surveys will be used as indicator variables. The model ensures diagnosis and follow-up treatment at no cost to the individual entirely through the tiered public health system of the state and country.

**Conclusions:**

Surveillance for NCDs is an essential facet of health care presently lacking in India. Atherosclerotic cardiovascular disease has a long gestation period in progression to the symptomatic phase of the disease, during which timely preventive and lifestyle measures would help prevent disease progression if implemented. Unfortunately, several asymptomatic individuals have never tested their plasma glucose, serum lipid levels, or blood pressure and are unaware of their disease status. Our model, implemented through the public health system using frontline health workers, would ensure individuals aged≥30 years at risk of disease are identified, and necessary lifestyle modifications and treatments are given. In addition, the surveillance at the community level would help create a general awareness of NCDs and lead to healthier lifestyle habits.

**Trial Registration:**

Clinical Trial Registry India CTRI/2018/07/014856; https://tinyurl.com/4saydnxf

**International Registered Report Identifier (IRRID):**

DERR1-10.2196/27299

## Introduction

Noncommunicable diseases (NCDs) are the leading cause of mortality and morbidity in India. Overall, NCDs account for 53% of deaths in India, predominantly due to cardiovascular diseases (CVD) [1,2]. The economic burden associated with CVD in India is enormous, with coronary artery disease, stroke, and diabetes estimated to have caused a cumulative income loss of US $233.6 billion in India between 2005 and 2015 [3]. The disease burden in absolute number is also rapidly increasing, and projections suggest that by the year 2030, there will be 101 and 218 million individuals in India with diabetes and hypertension, respectively [4-6]. The NCD epidemic in India is largely attributed to an aging population, rapid and unplanned urbanization, poor food habits, lack of exercise and sedentary lifestyles, environmental pollution, increased levels of stress, to name a few, along with tobacco and alcohol abuse [7-9].

Due to the rapid demographic and epidemiological transitions, NCDs have become the most important priority in health care planning in India [10,11]. However, the primary and secondary care facilities for early diagnosis, appropriate care, and ethical treatment for NCDs have not grown adequately. In contrast, tertiary services dominated by procedure-driven curative care have grown exponentially, especially in the private sector, considerably increasing out-of-pocket expenditures. This results in late detection, poor secondary prevention, resource-intensive treatment, and often the medical care being unaffordable and inaccessible to large sections of society [12,13].

In a previous epidemiology of noncommunicable diseases in rural areas (ENDIRA ) study of >100,000 individuals, poor awareness and control of NCDs were revealed, with 48% of individuals with abnormal plasma glucose values, 37% with high blood pressure, and 85% with high cholesterol values unaware of their disease status. In addition, the majority of newly diagnosed individuals had never checked these parameters prior, partly because of a feeling that all was well with them and also because there was no system in place for surveillance other than for a visit to a health care facility and consultation with a doctor [14-16].

Developing sustainable, context-specific, resource-sensitive, effective, and scalable health system models for disease surveillance, early detection, and secondary prevention strategies are crucial for planning NCD care in India and other low-resource settings. We aim to develop a model surveillance system for NCD risk factors and conditions at the community level and demonstrate the usefulness of a health system model to scale-up diagnosis, treatment, and effective follow-up of chronic NCD conditions in Kerala, India [17,18]. We describe the methodology of our model in detail in this manuscript.

## Methods

### Study Setting

The study will be conducted in 27 panchayats (lowest units in the three-tier system of local governance in rural India, often consisting of 25,000-40,000 people) and 3 municipalities (larger towns with a population between 100,000 and 1,000,000) of Ernakulam district, Kerala, South India. The study population consists of all permanent residents of the 27 villages and 3 towns above the age of 30 years. The approximate target population size is 600,000. Accredited social health activists (ASHAs) are voluntary health workers at the community level. In Kerala, each of them caters to a population of 1000 individuals [19].

### Surveillance System for NCD Risk Factors

We propose a surveillance system, which will involve active screening and diagnosis of NCD risk factors and conditions at the community level. The planned surveillance system is three-tiered: (1) doorstep community screening for blood pressure, weight, and blood glucose through ASHAs using point of care devices, (2) confirmation of test results for eligible individuals provided by ASHAs at the PHC level with borderline values for glucose, blood pressure, and weight or prediagnosed as having 1of the 5 identified major risk factor of atherosclerotic cardiovascular disease (ASCVD), namely diabetes, hypertension, tobacco use, dyslipidemia, or a family history of CVD and (3) a detailed evaluation for CVD, including a lipid profile, thyroid profile, and electrocardiogram, if required, in individuals with 2 or more of the conventional risk factors for ASCVD. At all levels, the ASHA facilitates the care and acts as a care coordinator for the respective individuals in her area. The screening process will be repeated every year, and a minimum of once in 3-month follow-up for all eligible individuals will be coordinated by the ASHAs.

### Design

#### Framework for a New Health System Model

**Figure 1:**
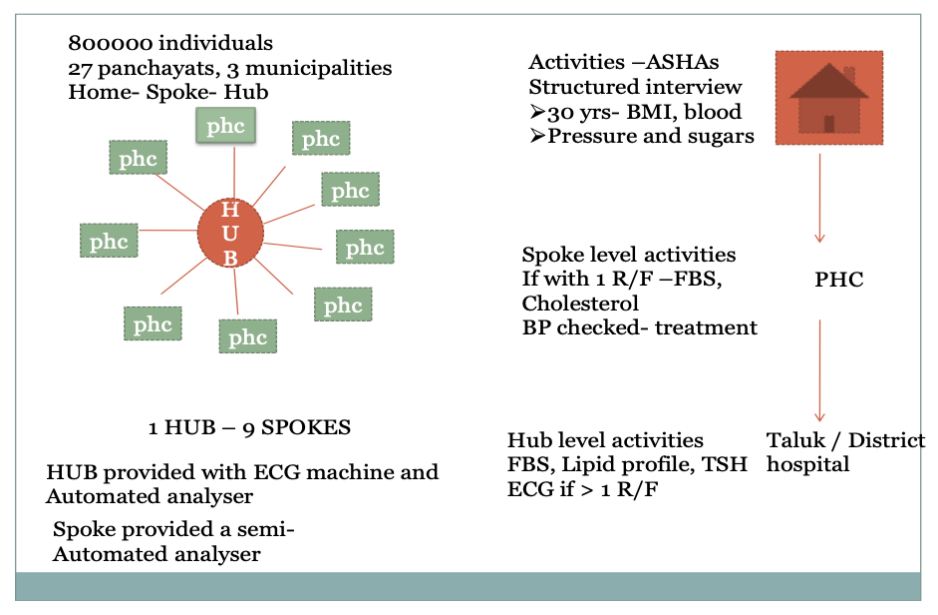
"Spoke and hub" model. ASHA: accredited social health activist; ECG: electrocardiogram; FBS: fasting blood sugar; PHC: primary health center; TSH: thyroid-stimulating hormone.

**Figure 2:**
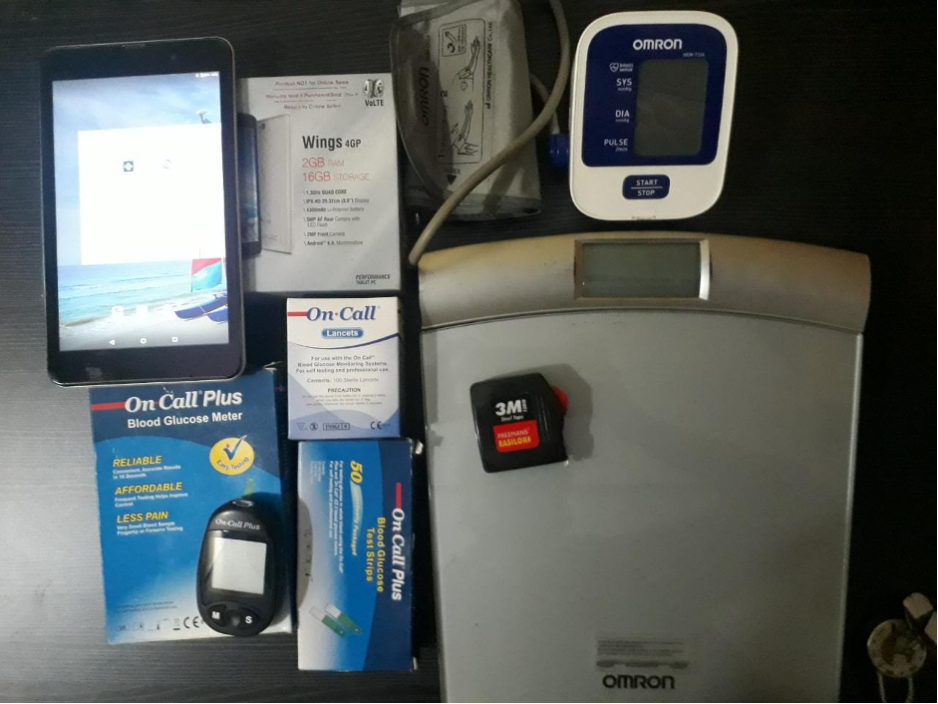
ASHA Kit includes 1 each of item: a tablet PC, glucometer, digital blood pressure apparatus, digital weighing scale, tape, lancets, and strips.

The proposed framework is of a “spoke and hub” model and is planned to be integrated into the existing public health care delivery system. As part of the Shraddha-Jagrithi project, a semi-automated blood analyzer will be provided to each primary health center (PHC; spokes) and a fully automated blood analyzer to the Taluk or district hospitals (hubs). In our model, one district hospital and 2 taluk hospitals will serve as hubs to spokes of 9 PHCs each under them (Figure 1). Trained ASHAs will conduct a detailed survey at the population level and cover all the houses in the selected area. The screening measurements and questionnaire administration for the survey will be done at the doorstep of all residents. The ASHA will administer a structured questionnaire (Multimedia Appendix 1) to capture relevant risk factor data from all study participants. ASHAs will use a tablet computer enabled with a GPS for data collection. Along with data, the device will capture the latitude and longitude of each household surveyed by the respective ASHAs. The ASHA workers will measure the weight and height of all adults aged above 30 years in the household according to standard methods described in the World Health Organization STEPwise approach to surveillance (STEPS) manual [20]. ASHAs will also measure random blood sugar from capillary blood using an ON CALL PLUS glucometer (ACON Labs Inc), and blood pressure will be measured using an OMRON HEM 7124 (OMRON Healthcare), automated digital blood pressure (BP) apparatus. BP readings will be taken twice (5 mins apart), and then an average of the 2 readings will be considered. Written informed consent will be obtained prior to all measurements (Figure 2).

A nodal officer and 2 study coordinators will coordinate the study. The study coordinators will also check data entry and veracity by contacting every 25th household. In addition, the study coordinators will calibrate the tools and troubleshoot the gadgets provided, namely glucometer and sphygmomanometer, at regular intervals. Individuals with surveillance parameters suggestive of a risk of diabetes, hypertension, obesity, current tobacco use, or with a family history of NCDs or ASCVD will be directed for more detailed risk stratification. We will use the following parameters in the estimation of risk for CVD: capillary fasting blood sugar values of >110 mg/dl, 2-hour postprandial value of >160mg/dl, or any random sugar value of >200mg/dl, a systolic blood pressure >140 mmHg, or a diastolic blood pressure >90 mmHg in isolation, or combination, a BMI >25 kg/m2, a family history of CVD in any first degree relatives of the individual (coronary, cerebrovascular, or peripheral artery disease) and tobacco use in any form over the past 30 days.

In case of a single risk factor, the detailed risk assessment will be done at the PHC (spoke hospital) located in the village of residence. Individuals with 2 or more of the risk factors for ASCVD will be directed to the hub center for further assessment, risk stratification, and treatment. In addition to the risk assessment, the NCD clinics of “hub” centers are entrusted with lifestyle interventions, including modules on tobacco and alcohol cessation, advice on dietary modification, and physical activity. Furthermore, medicines required to control NCD conditions as per the prevailing treatment guidelines will be provided at no cost to the patient by the NCD clinics in the PHCs.

The “hub and spoke” model will ensure the inclusion of diagnosis, evaluation, and treatment in the public health care system. The project provides the necessary equipment and instruments for analysis at the community, PHC, and taluk/district hospital tiers. The model will also ensure specialist care of disease conditions for eligible individuals at the hub centers. The PHCs are manned mainly by MBBS doctors, with very few having specialists, while the hub hospitals have specialist posts, with each of them having doctors trained in internal medicine and other specialties, including pediatrics and gynecology. The district National Health Mission and the ENDIRA investigators will provide the necessary training to ASHAs on surveillance methodology and entry and additional support to ASHAs and PHC physicians, with both patient advice and treatment and the modules on lifestyle interventions (eg, diet, exercise, tobacco cessation, etc).

#### Capacity Building as Part of the Shraddha-Jagrithi Project

As a part of the Shraddha-Jagrithi project, ASHAs will be given training on measuring the height and weight in individuals, blood sugar using a glucometer, blood pressure using the digital blood pressure apparatus, and entering gathered data on tablet computers with a GPS tracking facility. Separate training sessions will be conducted for each panchayat/municipality. In total, 30 full-day training sessions will be planned for training ASHAs at their respective PHCs/panchayats. The research team, including the investigators, nodal officer, and the project coordinators, will provide the necessary training. After explaining the project, ASHAs will be shown audio-visual modules on recording blood pressure, blood glucose, height, and weight. The audio-visual modules will be made available in the local language (Malayalam). Further, the ASHAs will be grouped into pairs and individually made to check blood glucose, blood pressure, and the height or weight of the partner ASHAs and vice versa.

The ASHAs will distribute pamphlets highlighting common NCDs and their preventive measures and enumerating dietary advice, exercise, cessation of tobacco, and alcohol, in the local language during the screening and measurement visits. In addition, the ASHAs will be primed regarding the primary prevention strategies and counseled on the possible questions that could be asked during those sessions. If ASHAs cannot address participants’ questions, they will be asked to connect directly with any study team member through the study coordinators.

## Results

### Evaluation of the Hub and Spoke Model

The model will be evaluated based on the utilization rate of various services offered at all tier levels. Indicator variables include the proportion of the target population screened, eligible individuals who reached the spoke centers for risk stratification, eligible individuals who received care at the hub, individuals with hypertension and diabetes who received care at the hub and spoke centers under either facility-level control or community-level control for hypertension and diabetes in annual surveys.

### Ethical Overview

The Institutional Ethics committee of Amrita Institute of Medical Sciences approved the Shraddha-Jagrithi project (IEC-AIMS-2017-CARD-459). Confidentiality of the data will be ensured as per the existing norms, and it will not be used for any kind of marketing or promotional programs. The data will be stored on a central server in the District hospital, Aluva. The study is registered under the Clinical Trial Registry India (registration number CTRI/2018/07/014856). Signed informed consent is taken from each participant by way of an electronic signature on the tablet PC in the local language.

### Statistical Analysis

Baseline characteristics will be explained separately for different strata (ie, by gender, socioeconomic status, and age groups). The differences across strata will be investigated using appropriate statistical tests. A two-sided *P* value <.05 will be considered statistically significant. The serial trend in the utilization rate of services offered and blood pressure and blood glucose control rates per the indicator variables will be checked by adopting multilevel modeling. We will describe the average pattern of change, difference in direction and rate of change, impact of covariates on differences in change pattern, and influence of circumstances over time on outcome variables. All analyses will be performed using SPSS statistical (version 18.0; IBM).

## Discussion

### Principal Findings

Our health system model for surveillance and management of NCDs at the community level will help reduce CVD incidence in the future. In addition, the model will help to achieve better control rates of blood pressure and blood glucose in individuals with hypertension and diabetes at the community level. Further, we expect that the framework proposed will help shift the population-level distribution of mean blood pressure and blood glucose towards the left of the distribution curve. This will have important implications in preventing future cardiovascular events.

Our framework will help us obtain reliable surveillance data on CVD risk factors at the population level regularly. It will also facilitate evaluating the impact of ongoing policy changes on screening, prevention, and control of CVD risk factors at the population level. A sound surveillance system is a prerequisite for effective control of chronic diseases and can significantly contribute to the planning and implementation of preventive measures. Although the public health system is grappling with resource constraints, there is room for more efforts to undertake systematic population-based chronic disease surveillance in India [21]. Reliable data on the prevalence of NCD risk factors is important to estimate the future burden of these risk factors or conditions. They are also important to identify and target subpopulations for effective preventive interventions [17,21]. We are trying to leverage the corporate-social responsibility (CSR) schemes for the public good of preventing and controlling CVD at the population level. Further, comprehensive NCD detection programs have been supported by corporates, with civil society leaders providing leadership [22]. We will also engage multiple stakeholders and involve political leaders and local public sector corporates in our CVD prevention and control efforts at the population level.

### Expected Outcomes

Our framework will be a significant first step in strengthening the public health system services in preventing and controlling CVDs at the population level. Our unique model of tapping CSR funds to prevent and control CVDs at the population level will be a benchmark for initiating such models in other settings across India. Our framework will also provide an opportunity to evaluate the impact of major policies on the prevention and control of CVDs at the population level.

## Appendix

Multimedia Appendix 1 Structured questionnaire.

## Abbreviations

ASCVD: atherosclerotic cardiovascular disease
ASHA: accredited social health activist
BP: blood pressure
CSR: corporate social responsibility
CVD: cardiovascular disease
ENDIRA: epidemiology of noncommunicable diseases in rural areas
NCD: noncommunicable disease
PHC: primary health center
