# Supplementary material for: A Sustainable Community-Based Model of Noncommunicable Disease Risk Factor Surveillance (Shraddha-Jagrithi Project): Protocol for a Cohort Study
Source: JMIR Res Protoc. 2021 Oct 22;10(10):e27299. doi: 10.2196/27299 (PMC8571687; doi:10.2196/27299)
Supplement: Multimedia Appendix 1 [file resprot_v10i10e27299_app1.pdf]

1) QUESTIONNAIRE – “SHRADDHA-JAGRITHI”

BASIC DETAILS:

1. Name of the Panchayat
2. Panchayat Reference Number
3. Subject Reference number
4. Household number
5. House name
6. Ward Number
7. House number
8. Religion
9. Above poverty line / Below poverty line

SOCIO-DEMOGRAPHIC PROFILE:

1. Name
2. Age in years
3. Sex
4. Education – school/graduate/post-graduate/doctorate
5. Occupation
6. Phone number – landline or mobile
7. Internet connectivity – yes / no and mode of connectivity – mobile/ PC / desk top
8. Income per month in Rupees a) < 10,000 b) 10000-30000 c) > 30,000

BEHAVIOURAL HABITS & DIET

1. Alcohol - Alcohol Yes /No amount per week
2. Do you smoke – Yes/No number per day
3. Do use other tobacco products – Yes/No
4. Do you exercise Yes/No, minutes per day
5. A) Type of oil used B) amount of oil used per month
6. A) Do you have red meats B) quantity bought per week C) quantity per head
7. Hours of sleep
8. Anxiety score – PHQ4

MEDICAL HISTORY

1. Hypertension – Yes/No, Since
2. Dyslipidemia- Yes/No, Since
3. Diabetes- Yes/No, Since, treatment – Diet/OHA/Insulin/Combination

4. Respiratory diseases- Yes/No, since
5. Surgeries undergone- Yes/No , Body part and surgery name
6. Snakebite- Yes/No
7. H/O Heart disease- Yes/No
8. H/O CVA- Yes/No, if yes – Stroke Questionnaire
9. Kidney disease-Yes/No, since

PHYSICO-BIOCHEMICAL PARAMETERS

1. Height in cm
2. Weight in kg
3. BMI
4. Systolic blood pressure in mm
5. Diastolic blood pressure in mm
6. Pulse rate per minute
7. Blood sugar - Random blood sugar/Fasting blood sugar/post-prandial blood sugar in mg%
8. Total cholesterol value in mg%

11) SURVEILLANCE KIT PROVIDED TO ASHAs

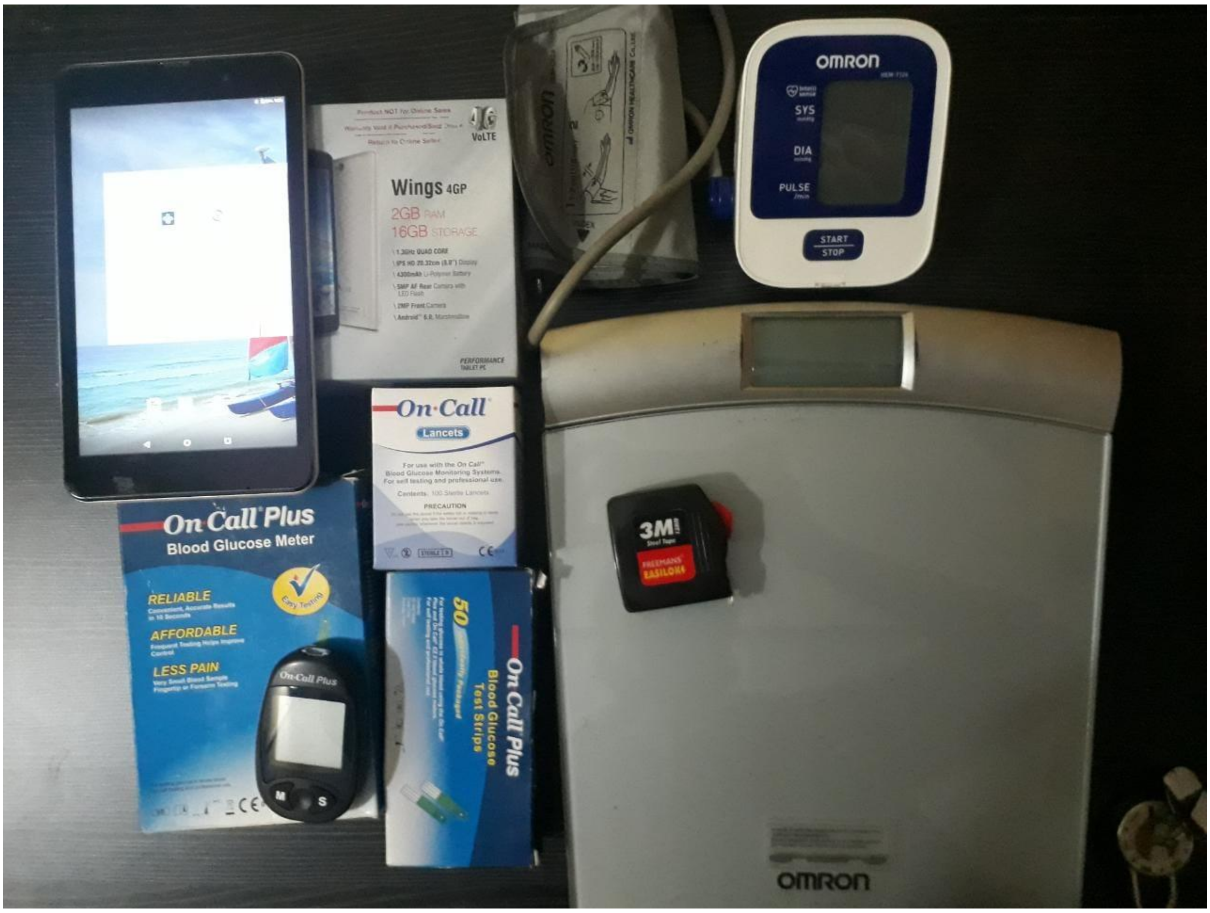

ASHA kit photograph Credit: Jaideep C Menon
